# Lived experience engagement in mental health research: Recommendations for a terminology shift

**DOI:** 10.1111/hex.13775

**Published:** 2023-05-11

**Authors:** Lisa D. Hawke, Natasha Y. Sheikhan, Faith Rockburne

**Affiliations:** ^1^ Centre for Addiction and Mental Health Toronto Canada; ^2^ University of Toronto Toronto Canada

**Keywords:** language, lived experience engagement, mental health, patient engagement, patient‐oriented research, substance use

Engaging people with lived experience (PWLE) of mental health challenges is increasingly considered a priority in health research settings.^1^ Lived experience engagement involves integrating PWLE in the full range of research processes, in roles such as advisors, collaborators, co‐researchers, or full partners. In these roles, PWLE can provide many contributions to research, from the earliest stages of identifying research questions to integrated and end‐of‐grant knowledge translation.^2^ There are published examples of PWLE engagement in a wide range of health research, across a diversity of study designs and research topics.^2^, ^3^, ^4^ Increasingly, engagement is being considered an ethical imperative and anti‐oppressive practice, given the history and continuing experience of inequities in both research and clinical practices.^5^ PWLE engagement is a pragmatic yet emancipatory research activity, according to which lived experience knowledge is valued to the same degree as the knowledge generated through empirical research.^6^

As standards for PWLE engagement continue to be established worldwide, numerous initiatives have been developed to guide its practice. In Canada, the Strategy for Patient‐Oriented Research (CIHR‐SPOR) defines and guides engagement.^7^ In the United States, the Patient‐Centered Outcomes Research Institute (PCORI) also provides guidance for engagement.^8^ ‘Patient and public involvement in research’ is another term under which direction is provided.^9^ Each of these spans all areas of health, including the full range of physical and mental health conditions. They also have in common the use of the term ‘patient’ to describe the people with whom we engage, which is the dominant term to describe engagement in mental health research.^4^

There are key differences to be noted in engagement across physical health versus mental health. Notably, mental health is a high priority area for engagement, due to past injustices and oppression specific to this sphere, such as paternalistic care cultures and a lack of recovery‐oriented care perspectives.^5^ Stigma toward mental health constitutes a barrier to effective engagement.^10^ An ‘us vs. them’ academic culture, skepticism, and mistrust in research in the face of past inequities in research and care emerge as additional challenges to overcome.^4^, ^11^ Issues related to cultural sensitivity and trauma‐informed approaches are relevant across health, but are particularly important in mental health, given the multifaceted and intersecting relationships between social determinants of health, trauma, mental health, and substance use. In addition, many mental health services are not publically funded in many jurisdictions, including our own, creating specific service access challenges that may intersect with the impacts of stigma. While representativeness is a challenge across health disciplines, it is especially important in mental health, which intersects with social determinants (e.g., stigma, social exclusion) that may sometimes affect the way PWLE engage with research. Adapting to working with individuals in the specific target audience within the extensive breadth of possible experiences across the mental health sphere (e.g., *lived experience of what type of mental health challenge?*), rather than targeting general lived experience of ‘mental health challenges’ as one homogeneous experience, becomes particularly important, yet is sometimes neglected. Together, these complex factors make engagement in mental health research unique.

In our experience working in mental health research, the term ‘patient’ consistently emerges as a barrier to authentic, meaningful, and respectful engagement. Indeed, through multiple discussions with a range of lived experience partners in different contexts, the term ‘patient’ has consistently been rejected, which has led to disruptions in the engagement process. In many research projects in mental health, the PWLE engaged are not expected to be currently registered patients in a psychiatric facility, which the term ‘patient’ suggests to them. PWLE have clearly expressed to us that ‘patient’ does not reflect the contributions they bring to projects as experiential experts, but rather reduces their status by putting them in a ‘sick’ role, perpetuating stigma. Notably, we have encountered situations in which the PWLE engaged did not understand that we were referring to them when using ‘patient,’ creating confusion in research discussions. We have also seen PWLE hesitate to be involved in projects or choose to withdraw to avoid the label ‘patient’ when submitting to a research funding stream specifically defined as emphasizing a ‘patient‐oriented’ research approach. PWLE have felt undervalued, disempowered, and disrespected by the term. As proponents and practitioners of engagement, it is our responsibility to address stigma, mitigate it, and create authentic, respectful working environments, which is not consistent with calling engaged PWLE partners ‘patients.’

As we have engaged PWLE in our research, we have navigated the challenge of language in part by negotiating language choices with the PWLE engaged. Consensus on language is difficult to achieve, as people have different roles and experiences in the mental health sphere. The sources of language preferences of PWLE are described in a conceptual model of diverse engagement, in relation to individuals' experiences of treatment and care.^12^ The model suggests that people with positive treatment and care experiences support the ‘patient’‐oriented language, while people with negative treatment and care experiences align with the ‘survivor’ discourse. ‘Consumer’ emerges as a preferred term for those with different constellations of treatment and care experiences. This illustrates the divergent opinions in language, which can be strong and are inherently linked to lived experience.

A variety of language choices have been suggested in our engagement activities. These include ‘people with lived experience,’ ‘peer,’ ‘experts by experience,’ and ‘consumer‐survivor,’ ‘advisor,’ or ‘educator,’ among others. For each term, there are proponents and dissenters. However, the term that has been most widely accepted by the people we engage with is ‘people with lived experience’ of mental health challenges. This is the term that, at the current time, resonates with the broadest spectrum of people with whom we engage in research processes. The term PWLE emphasizes lived experience, without aligning with the impacts of factors associated with the quality and experience of treatment and care. Consistent with recommendations in the literature, this is a person‐first language that puts the individual before the health condition.^13^, ^14^

It is important to balance the standardization of research terminology with flexibility in the terms we use. As per the guiding practices in engagement, it is imperative to talk to the people engaged in research to understand their preferences, including the terminology that they prefer we use to describe them. At the same time, the scientific literature needs consistent language for appropriate indexing to build a coherent evidence base, while funding bodies need to choose a term that will be clear to applicants. In the current state of PWLE preferences and the knowledge base, we, therefore, recommend ‘people with lived experience’, and derivatives such as lived‐experience engagement, lived‐experience research, lived‐experience partner, and lived‐experience advisor to replace patient‐based terms. This could stand alongside patient‐based terms when a broader scope of health is at play, but should be included when mental health is involved.

Language has the ability to stigmatize, but it can also legitimize, strengthen, and empower.^13^, ^15^ In mental health research, engagement practices invite PWLE of mental health challenges to lend their experiential expertise to enhance research practices. We call on researchers, funders, and other research support bodies to focus on the person, their experiences, and their expertise, not on the ‘sick’ role. To do so, we recommend that they use the term ‘people with lived experience’ when referring to engagement in mental health research, to better acknowledge the preferences, roles, and expertise of the people engaged in research endeavours.

## References

[1] Johnston JN , Ridgway L , Cary‐Barnard S , et al. Patient oriented research in mental health: matching laboratory to life and beyond in Canada. Res Involv Engagem. 2021;7(1):21. 33902751 10.1186/s40900-021-00266-1 PMC8074277

[2] Sangill C , Buus N , Hybholt L , Berring LL . Service user's actual involvement in mental health research practices: a scoping review. Int J Ment Health Nurs. 2019;28(4):798‐815. 30938019 10.1111/inm.12594

[3] Carroll P , Dervan A , Maher A , et al. Applying patient and public involvement in preclinical research: a co‐created scoping review. Health Expect. 2022;25(6):2680‐2699. 36217557 10.1111/hex.13615 PMC9700145

[4] Sheikhan NY , Kuluski K , Hebert M , Hawke LD . (under review). Exploring the impact of engagement in mental health research: a scoping review and content analysis. *Health Expect*. 10.1111/hex.13779 PMC10485342 37282732

[5] Pittaway E , Bartolomei L , Hugman R . ‘Stop stealing our stories’: the ethics of research with vulnerable groups. J Hum Rights Pract. 2010;2(2):229‐251.

[6] Allemang B , Sitter K , Dimitropoulos G . Pragmatism as a paradigm for patient‐oriented research. Health Expect. 2022;25(1):38‐47. 34748689 10.1111/hex.13384 PMC8849373

[7] Canadian Institutes of Health Research . Strategy for patient‐oriented research—patient engagement framework. 2019. Accessed March 10, 2023. https://cihr-irsc.gc.ca/e/48413.html

[8] Sheridan S , Schrandt S , Forsythe L , Hilliard TS , Paez KA . The PCORI engagement rubric: promising practices for partnering in research. Ann Fam Med. 2017;15(2):165‐170. 28289118 10.1370/afm.2042 PMC5348236

[9] Forbat L , Hubbard G , Kearney N . Patient and public involvement: models and muddles. J Clin Nurs. 2009;18(18):2547‐2554. 19207798 10.1111/j.1365-2702.2008.02519.x

[10] Ewalds Mulliez A‐P , Pomey M‐P , Bordeleau J , Desbiens F , Pelletier J‐F . A voice for the patients: evaluation of the implementation of a strategic organizational committee for patient engagement in mental health. PLoS One. 2018;13(10):e0205173. 30356239 10.1371/journal.pone.0205173 PMC6200221

[11] Worsley JD , McKeown M , Wilson T , Corcoran R . A qualitative evaluation of coproduction of research: ‘if you do it properly, you will get turbulence’. Health Expect. 2022;25(5):2034‐2042. 33949751 10.1111/hex.13261 PMC9615072

[12] Daya I , Hamilton B , Roper C . Authentic engagement: a conceptual model for welcoming diverse and challenging consumer and survivor views in mental health research, policy, and practice. Int J Ment Health Nurs. 2020;29(2):299‐311. 31538723 10.1111/inm.12653 PMC7328715

[13] Carroll SM . Respecting and empowering vulnerable populations: contemporary terminology. J Nurse Pract. 2019;15(3):228‐231.

[14] Jensen ME , Pease EA , Lambert K , et al. Championing person‐first language: a call to psychiatric mental health nurses. J Am Psychiatr Nurses Assoc. 2013;19(3):146‐151. 23698977 10.1177/1078390313489729

[15] Broyles LM , Binswanger IA , Jenkins JA , et al. Confronting inadvertent stigma and pejorative language in addiction scholarship: a recognition and response. Subst Abus. 2014;35(3):217‐221. 24911031 10.1080/08897077.2014.930372 PMC6042508

